# Cellular Dysfunction in Diabetes as Maladaptive Response to Mitochondrial Oxidative Stress

**DOI:** 10.1155/2012/696215

**Published:** 2012-01-02

**Authors:** Alba Naudi, Mariona Jove, Victoria Ayala, Anna Cassanye, Jose Serrano, Hugo Gonzalo, Jordi Boada, Joan Prat, Manuel Portero-Otin, Reinald Pamplona

**Affiliations:** Department of Experimental Medicine, Faculty of Medicine, University of Lleida-IRBLleida, 25008 Lleida, Spain

## Abstract

Oxidative stress has been implicated in diabetes long-term complications. In this paper, we summarize the growing evidence suggesting that hyperglycemia-induced overproduction of superoxide by mitochondrial electron transport chain triggers a maladaptive response by affecting several metabolic and signaling pathways involved in the pathophysiology of cellular dysfunction and diabetic complications. In particular, it is our goal to describe physiological mechanisms underlying the mitochondrial free radical production and regulation to explain the oxidative stress derived from a high intracellular glucose concentration and the resulting maladaptive response that leads to a cellular dysfunction and pathological state. Finally, we outline potential therapies for diabetes focused to the prevention of mitochondrial oxidative damage.

## 1. Introduction

The Diabetes Control and Complications Trial (DCCT) and the United Kingdom Prospective Diabetes Study (UKPDS) established that hyperglycemia is the initiating cause of the diabetic tissue damage which is verified clinically [1, 2]. Even though this process is modified by both genetic determinants of individual susceptibility and by independent accelerating factors such as hypertension, both the repeated acute changes in cellular metabolism and cumulative long-term changes in cellular constituents appear to be the mechanisms that mediate the cell-damaging effects of hyperglycemia.

The cell-damaging effects of hyperglycemia comprise the damage to a selective subset of cell types directly involved in diabetic complications: endothelial cells in the vascular system, mesangial cells in the kidney, neurons and neuroglia in the nervous system, and pancreatic *β* cells. Why are these cells especially vulnerable to hyperglycemic conditions? In the organism, most cells are able to downregulate the transport of glucose inside the cell when they are exposed to a hyperglycemic status, so that their intracellular glucose concentration stays constant. In contrast, the cells injured by hyperglycemia are those that cannot do this efficiently [3, 4], leading to high glucose levels inside the cell. In this scenario, available evidences demonstrate that a hyperglycemia-induced cellular oxidative stress is the basic mechanism underlying the physiopathology of the diabetic complications. Indeed it has been suggested that increased mitochondrial free radicals production during hyperglycemia may be central of the pathology of diabetes [5, 6]. Therefore, mitochondrial free radical production and oxidation-derived molecular damage may contribute to the onset, progression, and pathological consequences of diabetes. Here, we discuss how mitochondrial oxidative damage occurs, consider the maladaptive mechanisms by which it may contribute to the pathophysiology of diabetes, and outline potential therapeutic strategies to prevent it.

## 2. Physiology of the Mitochondrial Oxidative Damage

Inside mitochondria, electrons from reduced substrates move from complexes I and II of the electron transport chain through complexes III and IV to oxygen, forming water and causing protons to be pumped across the mitochondrial inner membrane. When glucose is metabolized through the tricarboxylic acid (TCA) cycle (or fatty acids through *β*-oxidation), it generates electron donors. The main electron donor is NADH, which gives electrons to complex I. The other electron donor generated by the TCA cycle is FADH_2_, formed by succinate dehydrogenase, which donates electrons to complex II. The proton motive force set up by proton pumping [7] drives protons back through the ATP synthase in the inner membrane, forming ATP from their precursors ADP (adenosine diphosphate) and phosphate [8]. The electron transport system is organized in this way so that the level of ATP can be precisely regulated.

In this context, a major side reaction is that electrons may leak from the respiratory chain and react with oxygen to form the free radical superoxide. Superoxide anion, the product of a one-electron reduction of oxygen, is the precursor of most reactive oxygen species (ROS) and a mediator in oxidative chain reactions [71–75]. So, oxygen reduction, needed for aerobic life, generates three main ROS, superoxide radical, hydrogen peroxide (H_2_O_2_), and hydroxyl radical. The hydroxyl radical can be generated by the combination of superoxide radical and H_2_O_2_ in the presence of traces of iron or copper during the Fenton-Haber-Weiss reaction. Thus H_2_O_2_, although it is not a free radical, can work as a Trojan horse, diffusing away from sites of ROS production to generate the hydroxyl and other reactive radicals at other cellular locations, hereby propagating oxidative damage. Other ROS of probable relevance for endothelial cells are the perhydroxyl radical, particularly near membranes where local pH is lower than in the bulk solution [76], singlet oxygen, and nitric oxide. In the case of mitochondria, nitric oxide production is much smaller than superoxide production. However, nitric oxide can still be important due to interaction with superoxide and other radicals to produce reactive nitrogen species like peroxynitrite [77], which can modify many kinds of macromolecules and possibly contribute to diabetes vascular complications [78].

Despite ROS can be generated at various sites and under various conditions (including, ischaemia-reperfusion, enzymatic reactions (e.g., the membrane NADPH oxidase, lipoxygenases, cyclooxygenases, peroxidases, and other heme proteins), the enzyme xanthine oxidase, peroxisomes, or the hepatic P-450 microsomal detoxifying system), in healthy cells under physiological conditions, most ROS are originated in mitochondria [79]. Currently, it is well known that mitochondrial ROS generation occurs at complex I [79–86] and at complex III [87, 88]. Concerning the electron transport component responsible for mtROS generation within complex I, flavin mononucleotide, ubisemiquinone species, or iron-sulphur clusters have been proposed [89–97].

The finding that the percentage of total electron flow directed to free radical generation in mitochondria is not constant in different tissues and different conditions inside a given tissue suggests that ROS generation is not a simple byproduct of mitochondrial respiration as is frequently assumed. Indeed there is a lack of stoichiometric coupling of ROS production to oxygen consumption [98]. Therefore, it should be better viewed as a homeostatically controlled variable.

**Figure 1:**
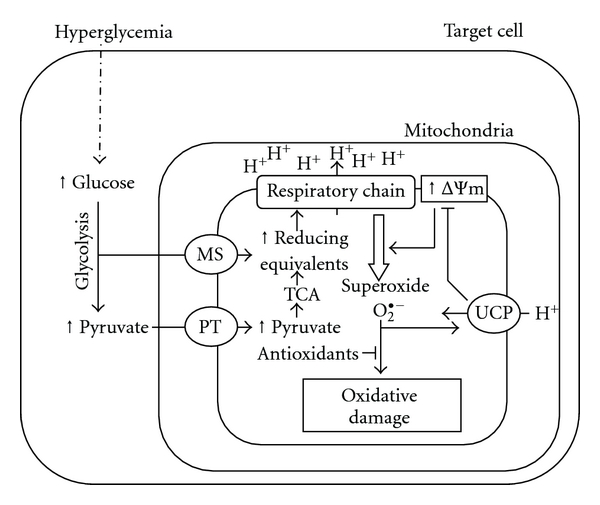
Uncoupling proteins (UCPs) respond to hyperglycemia-induced overproduction of mitochondrial superoxide by catalyzing mild uncoupling, which lowers membrane potential (ΔΨ*m*) and decreases superoxide production by mitochondrial complex I and III of the electron transport chain. Antioxidants limit the impact of superoxide production on molecular oxidative damage (for more details, see text). MS: mitochondrial redox shuttles; O_2_^•−^: superoxide radical; PT: pyruvate transporter; TCA: tricarboxylic acid cycle.

Are there physiological adaptation mechanisms with ability to modulate the rate of mitochondrial free radical generation? Available evidence seems to suggest that this is the case [99]. Among these adaptations, two negative feedback loops protect cells from ROS-induced damage. The first mechanism is characterized by regulation of uncoupling proteins (UCPs). During oxidation of substrates, the complexes of the mitochondrial electron transport chain reduce oxygen to water and pump protons into the intermembrane space, forming a proton motive force (Δp). However, some electrons in the reduced complexes also react with oxygen to produce superoxide. Superoxide can peroxidize membrane phospholipids, forming hydroxynonenal, which induces proton transport through the UCPs and the adenine nucleotide translocase. The mild uncoupling caused by proton transport lowers Δp and slightly stimulates electron transport, causing the complexes to become more oxidized and lowering the local concentration of oxygen; both these effects decrease superoxide production. Thus, the induction of proton leak by hydroxynonenal limits mitochondrial ROS production as a feedback response to overproduction of superoxide by the respiratory chain [89, 100, 101]. So, a possible antioxidant physiological function for UCPs has been proposed [100]. In this model, UCPs respond to overproduction of matrix superoxide by catalyzing mild uncoupling, which lowers proton motive force and would decrease superoxide production by the electron transport chain (Figure 1).

**Figure 2:**
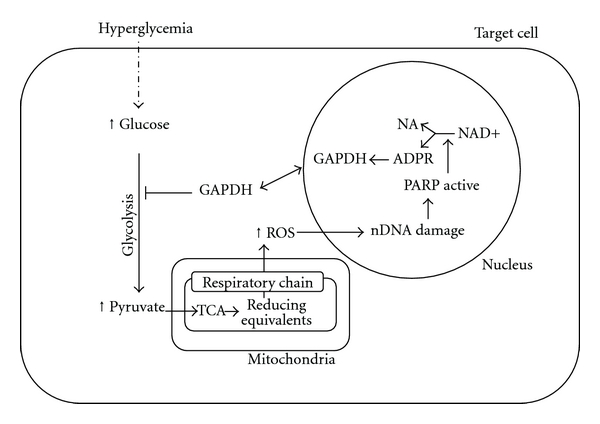
Hyperglycemia-induced mitochondrial free radical production induces DNA damage that activates PARP and modifies GADPH leading to a block of glycolysis (for more details, see text).

The second feedback loop consists of a regulation of the flux of metabolites to mitochondria. So, a transient overproduction of ROS by the mitochondrial electron transport chain can decrease the activity of the key glycolytic enzyme glyceraldehyde-3 phosphate dehydrogenase (GAPDH) by modifying the enzyme by ADP-ribosylation [102]. Poly(ADP-ribosyl)ation represents an immediate cellular response to DNA damage induced by oxidants [103–105]. In the absence of DNA single and double-strand breaks, poly(ADP-ribosyl)ation is a very rare event, but it can increase over 100-fold upon DNA damage. Under these conditions, about 90% of poly(ADP-ribose) is synthesized by poly(ADP-ribose) polymerase 1 (PARP-1). PARP-1 is constitutively expressed but enzymatically activated by DNA strand breaks. So, PARP-1 functions as a DNA damage sensor and signaling molecule binding to both single- and double-stranded DNA breaks. It catalyses the formation of ADP-ribose from the oxidized form of nicotinamide adenine dinucleotide (NAD+) by cleavage of the glycosidic bond between nicotinamide and ribose. Glutamate, aspartate, and carboxyterminal lysine residues of target (“acceptor”) proteins are then covalently modified by the addition of an ADP-ribose subunit, via formation of an ester bond between the protein and the ADP-ribose residue. So, poly(ADP-ribosyl)ation is a covalent posttranslational protein modification linked with genome protection [103, 106]. In this scenario, it is plausible to suggest that the inhibitory effect of ADP-ribosylation on GAPDH probably represents a feedback loop in order to reduce levels of glycolysis and transiently block the subsequent flux of metabolites to mitochondria allowing a decrease in the levels of reducing equivalents and the subsequent mitochondrial ROS production and oxidative cellular molecular damage (Figure 2).

## 3. Mitochondrial Antioxidant Defenses

Oxidative stress homeostasis (e.g., balance between ROS production and elimination) relies on endogenous cellular antioxidants [99, 107–109]. Mitochondria, from an intracellular organelle comparative approach, are endowed with the best antioxidants, detoxifying and repair systems against oxidative damage. So, the antioxidant enzyme MnSOD (manganese superoxide dismutase) converts superoxide to H_2_O_2_. The mitochondrial isoform of glutathione peroxidase (GPx) and the thioredoxin-dependent enzyme peroxiredoxin III both detoxify H_2_O_2_; alternatively, H_2_O_2_ can diffuse from the mitochondria into the cytoplasm. The mitochondrial glutathione (GSH) pool is different from that in the cytosol and is maintained in its reduced state by a mitochondrial isoform of glutathione reductase (GR). This enzyme requires NADPH, which is produced within mitochondria by the NADP-dependent isocitrate dehydrogenase and through a proton electrochemical potential gradient-dependent transhydrogenase. Within the mitochondrial phospholipid bilayer, the fat-soluble antioxidants vitamin E and coenzyme Q (CoQ) both prevent lipid peroxidation, while CoQ also recycles vitamin E and is itself regenerated by the respiratory chain. The mitochondrial isoform of phospholipid hydroperoxide glutathione peroxidase [110] degrades lipid peroxides within the mitochondrial inner membrane. There are also a variety of specific mitochondrial mechanisms to repair or degrade oxidatively damaged lipids [108, 110], proteins [111], and mtDNA [112].

## 4. Hyperglycemia Induces Permanent Overproduction of Superoxide by Mitochondrial Electron Transport Chain

As mentioned above, the major sites of ROS generation are the complexs I and III of the mitochondrial electron transport chain. In cells under sustained high glucose concentrations, there is more glucose being oxidized in the TCA cycle. This situation drives to pushing more electron donors (NADH and FADH_2_) into the electron transport chain thus leading to an increase in ROS generation [5, 6]. This is so because in this situation, there is a higher degree of reduction of complexes I and III increasing their rate of ROS production. The rate of mitochondrial ROS generation strongly increases with a sigmoidal kinetics when the NADH/NAD+ ratio is increased, because this dramatically increases the degree of reduction of the complex I ROS generator [84, 98]. In an identical way, in the insulin resistance syndrome, there is an increased free fatty acid (FFA) flux from adipocytes into arterial endothelial cells that might result in increased FFA oxidation by the mitochondria. Since both *β*-oxidation of fatty acids and oxidation of FFA-derived acetyl CoA by the TCA cycle generate the same electron donors (NADH and FADH_2_) generated by glucose oxidation, increased FFA oxidation may cause mitochondrial overproduction of ROS [113] by exactly the same mechanism described above for hyperglycemia, and in both cases can be reversed upon exposure to agents that act as mitochondrial uncouplers or electron transport chain inhibitors.

Concomitantly with the hyperglycemia-induced mitochondrial free radical overproduction, it has been described that in hyperglycemia Ucp2 gene transcription is activated by key regulatory proteins such as peroxisome proliferator-activated receptors (PPARs), forkhead transcription factors, sterol regulatory element-binding protein-1c (SREBP-1c) [114], and AMP-activated protein kinase [115]. Additionally, the pathological and persistent overproduction of ROS by the mitochondrial electron transport chain decreases the activity of the key glycolytic enzyme GAPDH. The inhibition of GAPDH activity by “hyperglycemia” does not occur when mitochondrial overproduction of superoxide is prevented by either UCP1 or MnSOD [116]. In addition, subsequent studies demonstrate that persistent high intracellular glucose concentration-induced superoxide inhibits GAPDH activity in vivo by modifying the enzyme by ADP-ribosylation [102]. By inhibiting mitochondrial superoxide production with either UCP-1 or MnSOD, it prevented the modification of GAPDH by ADP-ribose and the reduction of its activity. Most importantly, the modification of GAPDH is prevented by a specific inhibitor of poly(ADP-ribose) polymerase (PARP), the enzyme that makes these polymers of ADP-ribose, establishing a cause-and-effect relationship between PARP activation and the changes in GAPDH [5]. Therefore, this mechanism seems to indicate that the stress-induced block of glycolysis is not the result of a passive oxidative damage but rather an active cell adaptation programmed via ADP-ribosylation for cell self-defence.

However, the chronic increase in target cells of the intracellular glucose concentration and permanent block of glycolysis leads to a maladaptive response derived from the upstream accumulation of glycolytic metabolites which are substrates for the activation of metabolic pathways involved in the development of diabetic complications. In addition to this maladaptive response, the block of glycolysis leads to a fall of mitochondrial substrates that originates a reduced mitochondrial energy production and subsequent cell exhaustion that can be a determinant element in the endothelial cell dysfunction. In this scenario, other cellular sources of free radical generation could take the relief to mitochondria assuming a relevant role in a potential second round of cellular oxidative molecular damage.

## 5. Hyperglycemia-Induced Mitochondrial Free Radical Generation Activates Damaging Downstream Cellular Pathways

From the scenario described above, it was proposed that different pathogenic mechanisms leading to the development of diabetic complications do reflect a single hyperglycemia-induced process [5]. This process is based on that hyperglycemia, through the overproduction of free radicals by the mitochondrial electron transport chain, decreases the activity of the key glycolytic enzyme GAPDH. So, when GAPDH activity is inhibited, the level of all the glycolytic intermediates located upstream of GAPDH increases. Increased levels of the upstream glycolytic metabolite glyceraldehyde-3-phosphate activate two pathogenic pathways: (a) it activates the glycation pathway because methylglyoxal, a glycation precursor, is formed from glyceraldehyde-3 phosphate [117–119], and (b) it also activates the protein-kinase C pathway because diacylglycerol, one of its activators, is also formed from glyceraldehyde-3 phosphate [102, 120]. Further upstream, levels of the glycolytic metabolite fructose-6 phosphate increase, which increases flux through the hexosamine pathway, where fructose-6 phosphate is converted by the enzyme GFAT to UDP-*N*-acetylglucosamine (UDP-GlcNAc) increasing the chances for hexosamine modification of proteins [116]. Finally, inhibition of GAPDH increases intracellular levels of the first glycolytic metabolite, glucose. This increases flux through the polyol pathway, where the enzyme aldose reductase reduces it, consuming NADPH in the process and reducing available GSH [120–123].

**Figure 3:**
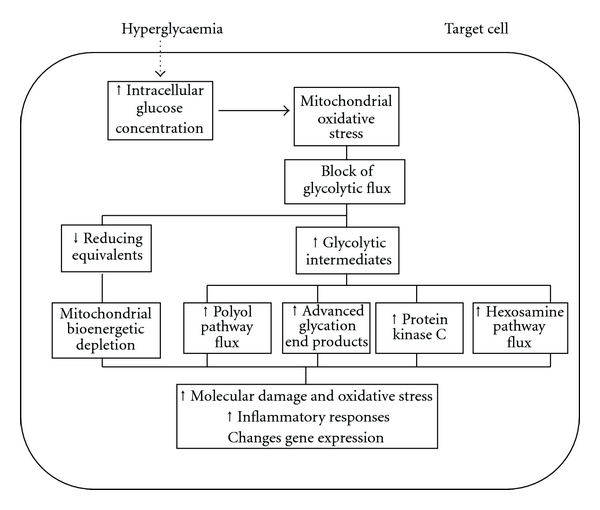
Intracellular high-glucose metabolism and oxidative stress. When intracellular glucose concentration increases in target cells of diabetes complications, it causes increased mitochondrial production of ROS and activates negative feedback loops to protect target cells from ROS-induced damage. The maladaptive response, however, leads to the activation of metabolic pathways that are involved in the diabetes vascular disfunction.

 Besides these maladaptive damaging cellular pathways, it must be considered the cellular responses derived from the PPAR overactivation as important mechanism of tissue damage also leading to an endothelial dysfunction in diabetic blood vessels, which importantly contributes to the development of various diabetic complications. Thus, PPAR activation, in addition to the mitochondrial bioenergetic depletion due to the block of glycolysis, potentiates in a maladaptive process the expression of various proteins at the transcriptional level [124]. Of special importance is the regulation by PARP-1 of the production of inflammatory mediators such as inducible nitric oxide synthase (iNOS), intercellular adhesion molecule-1 (ICAM-1), and major histocompatibility complex class II. NF-*κ*B is a key transcription factor in the regulation of this set of proteins, and PARP has been shown to act as a coactivator in the NF-*κ*B-mediated transcription. Poly(ADP-ribosyl)ation can loosen up the chromatin structure, thereby making genes more accessible for the transcriptional machinery [118]. Therefore, all these metabolic pathways originate alterations in gene expression, inflammatory responses, and structural and functional changes in cellular constituents that also participate in the molecular basis of the vascular diabetic process (Figure 3).

## 6. Protein Oxidative Damage: Protein Carbonyl Content in Diabetes

Oxidative damage occurs whenever the ROS produced by mitochondria evade detoxification, and the steady-state level of molecular oxidative damage depends on the relative rates of damage accumulation, repair, and degradation. ROS can damage all types of biomolecules, and oxidative damage to DNA, lipids and proteins can be deleterious and concomitant [107]. The primary cellular target of oxidative stress depends upon the cell type, the nature of the stress imposed, the susceptibility to oxidation of the target molecule, the site of generation, the proximity of ROS to a specific target, and the severity of the stress. In this context, protein oxidation demands an especial mention because proteins constitute the major “working force” for all forms of biological work. Furthermore, their exact conformation and pattern of folding are tightly related to their activity and function. So, the consequent loss of function and structural integrity of modified proteins can have a wide range of downstream functional consequences and may be the cause of subsequent cellular dysfunctions and tissue damage (Table 1). The products of oxidation of amino acids are indicators of modification to proteins in biological systems [125–129]. They include oxidized amino acids, modified amino acids by reactive nitrogen species and chlorination reactions, and crosslinks formed by a combination of enzymatic and nonenzymatic mechanisms.

Amino acid residues in proteins are highly susceptible to oxidation by one or more reactive species. Many different types of protein oxidative modification can be induced directly by ROS or indirectly by reactions of secondary byproducts of oxidative stress (basically derived from the oxidation of both carbohydrates and polyunsaturated fatty acids that lead to the formation of the named reactive carbonyl species, RCOs [130]). Cysteine and methionine are particularly prone to oxidative attack by almost all ROS. Protein modifications are elicited by direct oxidative attack on Lys, Arg, Pro, or Thr, or by secondary reaction of Cys, His or Lys residues with reactive carbonyl compounds can lead to the formation of protein carbonyl (PCO) derivatives (aldehydes and ketones) [125, 130, 131] (Table 2).

Glutamic semialdehyde is a product of oxidation of arginine and proline, and aminoadipic semialdehyde, of oxidation of lysine. They account for 55–100% of the total carbonyl value in several metal ion-catalyzed oxidation (MCO) systems [128, 132]. Sensitive gas chromatography-mass spectrometry based analytical methods has allow their quantitation in a variety of biological samples providing specific information on the oxidative status of proteins that is complementary to that afforded by protein carbonyls, and will be useful tools in the ongoing effort to define and assess the role of protein oxidation in diabetes complications [95, 132].

Other oxidation-derived protein damage markers include protein modifications derived from reactive nitrogen species (RNS). Nitric oxide generated from nitric oxide synthetases plays an important role in the regulation of various physiological parameters (very especially at the vascular level) but due to its free radical nature, it could also react with superoxide radical to form highly reactive peroxynitrite functions [133]. It has been established that aromatic amino acids, cysteine, and methionine residues of proteins are particularly sensitive to modification by RNS. These reactions lead to nitration of tyrosine residues of proteins [134, 135], the oxidation of methionine residues to methionine sulfoxide, and the nitrosation of protein sulfhydryl groups to RSNO derivatives [136–138].

Studies of the formation of PCOs cannot differentiate between those produced through direct protein oxidation and those formed by the addition of previously oxidized molecules, and hence protein carbonyl content (PCC) must be considered as a broad and unspecific marker of oxidation. Because carbonyls are relatively difficult to induce compared with, for example, methionine sulphoxide and cysteinyl derivatives, they might indicate a more rigorous oxidative stress. Indeed, elevated levels of PCC are generally a sign not only of oxidative stress, but also of disease-derived protein dysfunction. PCC can have an advantage over both carbohydrate and lipid oxidation products as markers of oxidative stress; oxidized proteins are generally more stable.PCCs form early and circulate in the blood for longer periods (their elevation in serum is stable for at least four hours), compared with other parameters of oxidative stress, such as glutathione disulphide and malondialdehyde [131]. The PCC seems to be a common phenomenon during oxidation-derived protein damage, and their quantification can be used to measure the extent of chemical and nonenzymatic oxidative modification. This has driven the development of various sensitive but unspecific biochemical (spectrophotometric and fluorometric) and immunological (western blot, enzyme-linked immunosorbent assay (ELISA), and proteomics) methods for the detection and measurement of the PCC in tissues and body fluids; in all of them 2,4-dinitrophenylhydrazine is allowed to react with the PCOs to form the corresponding hydrazone, which can be analyzed by the above mentioned methods. Currently, PCC is the most general indicator and by far the most commonly used marker of protein oxidation. Because the mechanisms of PCC generation are nonspecific, it has been argued that other protein modifications, such as the conversion of tyrosine residues to 3-chlorotyrosine, 3-nitrotyrosine or dityrosine, arginine and proline to glutamic semialdehyde, or lysine to aminoadipic semialdehyde, are better markers of oxidative stress. However, the tissue levels of such markers are orders of magnitude lower than the overall PCC and, hence, their measurement often requires highly sensitive and expensive methods such as mass spectrometry [109, 130, 132].

 Tables 3 and 4 summarize available studies where PCC was analyzed by different methods in the diabetic status. From this summary of the effects of diabetes on PCC, it is possible to propose some general ideas: (1) Mouse, rabbit, and especially rat are the animal species used as reference for the study of the effects of experimental diabetes, being the STZ-induced diabetes the experimental model predominantly, but not exclusively, used. (2) PCC levels are consistently increased in all the analyzed tissues independently of the analytical method used. Of particular interest are the increased PCC levels showed by the organs containing the selective subset of cell types directly involved in diabetic complications: vascular system, kidney, brain, and pancreas. (3) In humans, most studies are focused to Type 2 diabetes and the measurement of PCC in plasma proteins. (4) In humans, elevated PCC levels have been detected in both Type 1 and Type 2 diabetes. (5) Plasma PCC levels are significantly higher in diabetic children and adolescents without complications compared with control subjects, suggesting that oxidative protein damage occurs at the onset of disease and tends to increase in the later stages. (6) The presence of a diabetic complication is associated with higher PCC levels. (7) There is a lack of studies specifically driven to the vascular system.

## 7. Current Antioxidant Therapeutic Strategies

Hyperglycemia-induced overproduction of superoxide by mitochondrial electron transport chain induces a cellular maladaptive response that triggers several metabolic pathways of injury involved in the endothelial dysfunction and contributes to the progressive development of micro- and macrovascular complications and multiorgan damage. Consequently, inhibition of mitochondrial oxidant generation and/or oxidative-derived molecular damage might provide a potential approach for the prevention of diabetic vascular complications.

 Even though it is well established that good (but strict) glycemic control is the basis for the prevention of diabetic complications, there is no doubt that preventive measures targeting other risk factors should be also achieved. Therapeutic strategies for diabetic vascular complications should consist in the modulation of afflicted pathways. Thus, therapeutic strategies to limit mitochondrial radical production during hyperglycemia and to counteract their damaging effects could be useful complements to conventional therapies designed to normalize blood glucose. As our understanding of molecular mechanisms evolves, it is becoming clear that a more comprehensive approach is needed. Based on the numerous evidence of a role of oxidative stress in the pathogenesis of vascular complications, the use of for example, antioxidants, uncouplers, or PARP inhibitors should represent an appealing approach. Candidate “drugs” include: vitamins A, C, and E, alpha-lipoic acid, SOD and catalase mimetics, L-propionyl carnitine, taurine, acetyl-L-carnitine, U83836E (a ROS scavenger), M40403 (a manganese superoxide dismutase mimetic), PKC-b inhibitors, peroxynitrite catalyst FP15, mitochondrial uncoupler DNP, PARP inhibitors, transketolase inhibitors, melatonin, statins, angiotensin converting enzyme inhibitors, angiotensin II receptor blockers, thiazolidinediones, synthetic pyridoindole antioxidant stobadine (STB), extracts from different natural sources (e.g., Artemisia campestris, Centaurium erythraea), the metal chelator pyrrolidine dithiocarbamate (PDTC), and plant polyphenols (e.g., myricetin), among others.

PARP inhibition may emerge as a novel approach for the prevention or reversal of diabetic complications. The benefits and potential risks associated with chronic administration of PARP inhibitors are discussed in a recent review [139]. The comparative therapeutic utility of PARP inhibition for the experimental therapy of diabetic complications should be explored by additional preclinical and subsequent clinical investigations. The development of uncoupling strategies is not forthcoming [93]. So, the time is upon us to test antioxidant therapies in diabetes [78, 93].

## 8. Conclusions

Hyperglycaemia is the first trigger in the pathogenesis of diabetic vascular complications and it activates many metabolic pathways and their downstream mediators. Several mitochondrial and other intracellular pathways are implicated in the increased production of oxidants. In subjects with diabetes, oxidative damage is enhanced and contributes to the development of endothelial dysfunction and vascular complications. Nevertheless, there still is a considerable wealth of knowledge to be acquired, concerning oxidative stress and diabetes. Assuming that oxidative stress has also a signalling role (exceeding the role of NO), how the signaling role of oxidative stress is modified by diabetic status is still an open question. It needs to be elucidated how the general increase of protein oxidative damage has an impact on the signalling modules of oxidative stress. Furthermore, with a wide knowledge on protein oxidative modification chemistry, there is still lacking a comprehensive study dissecting the potential pathways of protein oxidative modifications in diabetes and diabetes complications. Numerous antioxidant agents are being investigated and there is growing interest in developing new compounds specifically targeting oxidative stress. However, up to now, there is a lack of supporting evidence for an extensive use of antioxidants for preventing or treating diabetic vascular complications. A better and more precise knowledge of the molecular mechanisms underlying hyperglycaemia-related damage will help in developing better therapies. When the answer of these and other relevant questions will be available, then a rationale intervention on ROS homeostasis, more directed than the mere supplementation with antioxidants, will be granted for therapy of diabetes vascular complications.

## Figures and Tables

**Table 1:** Effects of oxidative damage in protein structure and function.

(i) Cleavage of peptide bonds	
(ii) Direct reaction of proteins with ROS can lead to formation of protein derivatives or peptide fragments possessing highly reactive carbonyl groups (ketones, aldehydes)	
(iii) Formation of intra- or interprotein cross-linked derivatives that can lead to the formation of aggregates by (a) direct interaction of two carbon-centered radicals; (b) interaction of two tyrosine radicals; (c) oxidation of cysteine sulfhydryl groups; (d) interactions of the carbonyl groups of oxidized proteins with the primary amino groups of lysine residues in the same or a different protein; (e) by noncovalent interactions such as hydrophobic and electrostatic interactions between oxidized residues	
(iv) Partial unfolding or denaturation with a concomitant increase in surface hydrophobicity	
(v) Loss of function (e.g., enzyme activity)	

**Table 2:** Markers of oxidative damage in proteins.

Amino acid	Product
(i) Arginine	Glutamic-semialdehyde
(ii) Cysteine	Cysteine disulfides, Sulfenic acid
(iii) Histidine	Aspartate Asparagine 2-Oxoimidazoline 2-Oxohistidine
(iv) Leucine	3-,4-,5-Monohydroxyleucine
(v) Leucine, valine, isoleucine, proline, and others	Protein carbonyls
(vi) Lysine	2-Amino-adipic-semialdehyde
(vii) Methionine	Methionine sulfoxide
(viii) Phenylalanine	ortho- and meta-tyrosine
(ix) Proline	Glutamate
	Glutamic-semialdehyde
	2-Pyrrolidone
	4-,5-Hydroxyproline
	Pyroglutamic acid
(x) Threonine	2-Amino-3-ketobutyric acid
(xi) Tryptophan	2-, 4-, 5-, 6-, or 7-OH tryptophan N-formylkynurenine Kynurenine
(xii) Tyrosine	Di-tyrosine (Tyr-Tyr cross-links) Dihydroxyphenylalanine (DOPA) 3-Nitrotirosine 3-Chlorotyrosine

**Table 3:** Effects of experimental diabetes in levels of protein carbonyls.

Tissue	Model	Effect	Reference
*Mouse*			
Aorta	*BKS.cg-m +/+ Lepr db/J mice versus wild type*	*↑*	[9]
Hippocampus and cerebral cortex	*Streptozotocin*	*↑*	[10]
Kidney	*Type 2 diabetic db/db versus normoglycemic wild type mouse *	*↑*	[11]
Lenses	*Streptozotocin*	*↑*	[12]
*Rat*			
Aorta	*Goto-Kakizaki rats*	*↑*	[13]
Bone	*Goto-Kakizaki rats*	*↑*	[14]
Brain	*Galactose-induced hyperglycemia*	*↑*	[15]
Brain	*Goto-Kakizaki rats*	*↑*	[16]
Brain	*Streptozotocin*	*=*	[17]
Heart	*Streptozotocin*	*↑*	[18]
Heart	*Streptozotocin*	*↑*	[19]
Heart	*Streptozotocin*	*↑*	[20]
Heart	*Streptozotocin*	*↑*	[21]
Heart	*Streptozotocin*	*↑*	[17]
Hemoglobin	*Streptozotocin*	*↑*	[22]
Intestinal tissue	*Streptozotocin*	*↑*	[23]
Kidney	*Streptozotocin*	*↑*	[24]
Kidney	*Streptozotocin*	*↑*	[18]
Kidney	*Zucker obese hyperglycemic rats (ZDFn Gm-fa/fa)*	*↑*	[25]
Kidney	*Streptozotocin*	*↑*	[26]
Kidney	*Streptozotocin*	*↑*	[17]
Lens proteins	*Streptozotocin*	*↑*	[27]
Liver	*Streptozotocin*	*↓*	[24]
Liver	*Pregnant diabetic rats versus control rats*	=	[28]
Liver	*Galactose-induced hyperglycemia*	*↑*	[15]
Liver	*Streptozotocin*	*↑*	[18]
Liver	*Streptozotocin*	*↑*	[29]
Liver	*Streptozotocin*	*↑*	[23]
Liver	*Streptozotocin*	*↑*	[30]
Liver	*Streptozotocin*	*↑*	[17]
Lung	*Streptozotocin*	*↑*	[31]
Pancreas	*Streptozotocin*	*↑*	[18]
Pancreas	*Alloxan*	*↑*	[32]
Pancreas	*Streptozotocin*	*↑*	[17]
Pancreas	*Streptozotocin*	*↑*	[33]
Plasma proteins	*Streptozotocin*	*↑*	[34]
Plasma proteins	*Streptozotocin*	*↑*	[35]
Plasma proteins	*Streptozotocin*	*↑*	[36]
Red blood cells	*Streptozotocin*	*↑*	[18]
Retinal Müller cells	*Streptozotocin*	*↑*	[37]
Skeletal muscle	*Glupreclamp infusion versus control*	*↑*	[38]
Skeletal muscle	*Otsuka Long Evans Tokushima Fatty (OLETF) rats versus LETO rats*	*↑*	[39]
Skeletal muscle (Soleus muscles)	*Goto-Kakizaki rats*	*↑*	[13]
Skeletal muscle (Plantaris muscle)	*Obese Zucker rats versus lean Zucker rats *	*↑*	[40]
Skeletal muscle	*Streptozotocin*	*↑*	[41]
Testis and epididymal sperm	*Streptozotocin*	*↑*	[42]
Vascular smooth muscle cells	*Glucose incubation*	*↑*	[43]
*Rabbit*			
Heart	*Alloxan*	*↑*	[44]
Lens proteins and cells	*In vitro incubation*	*↑*	[45]

**Table 4:** Effect of diabetes in protein carbonyl content (PCC) levels from human tissues.

Tissue	Model/condition	Effect	Reference
Erythrocytes	*Obese type 2 diabetic patients*	*↑*	[46]
Erythrocytes	*Type 2 diabetic patients versus healthy subjects*	*↑*	[47]
Erythrocyte membrane	*Type 2 diabetic patients versus healthy subjects*	*↑*	[48]
Lymphocytes	*Type 2 diabetic patients versus age-matched controls*	*↑*	[49]
Lymphocytes	*DM patients versus healthy subjects*	*↑*	[50]
Placenta	*Women with gestational diabetes versus healthy pregnant women*	*↑*	[51]
Plasma proteins	*Type 2 diabetic patients versus healthy subjects*	*=*	[52]
Plasma proteins	*Dialysis patients versus control subjects*	*↑*	[53]
Plasma proteins	*Diabetic type 2 patients versus healthy subjects*	*↑*	[54]
Plasma proteins	*Type 1 diabetes without complications*	*↑*	[55]
Plasma proteins	*Type 1 diabetes with complications*	*↑*	[55]
Plasma proteins	*Chronic kidney disease patients versus healthy subjects*	*↑*	[56]
Plasma proteins	*Diabetes type 2 versus healthy subjects*	*↑*	[57]
Plasma proteins	*Diabetes type 2 associated with CVD versus healthy subjects*	*↑*	[57]
Plasma proteins	*Good glycemic control versus poor glycemic control*	*↑*	[58]
Plasma proteins	*Type 1 diabetic patients*	=	[59]
Plasma proteins	*End-stage renal disease*	*↑*	[59]
Plasma proteins	*Heart failure + diabetes versus healthy subjects*	*↑*	[60]
Plasma proteins	*Type 2 diabetes without microangiopathy versus healthy subjects*	*↑*	[61]
Plasma proteins	*Type 2 diabetes with microangiopathy versus healthy subjects*	*↑*	[61]
Plasma proteins	*Type 2 diabetic patients versus age-matched controls*	*↑*	[49]
Plasma proteins	*Childhood type 1 diabetes*	*↑*	[62]
Plasma proteins	*Diabetic patients without ulcer versus healthy subjects*	*↑*	[63]
Plasma proteins	*Diabetic patients with foot ulcer grade 1 versus healthy subjects*	*↑*	[63]
Plasma proteins	*Diabetic patients with foot ulcer grade 2 versus healthy subjects*	*↑*	[63]
Plasma proteins	*Diabetic patients versus healthy subjects*	*↑*	[64]
Plasma proteins	*IGT subjects versus healthy subjects*	*↑*	[64]
Plasma proteins	*Diabetic type 2 patients versus healthy subjects*	*↑*	[65]
Platelets	*Type 2 diabetes (young versus elderly)*	*↑*	[66]
Serum	*Type 1 diabetic patients versus healthy subjects*	*↑*	[67]
Serum	*Diabetic patients versus healthy subjects*	*↑*	[68]
Serum	*Diabetic nephropathy patients versus healthy subjects*	*↑*	[68]
Skin collagen	*Type 2 diabetes*	*↑*	[69]
Subretinal fluid	*Diabetic patients versus control subjects*	*↑*	[70]
